# Importance of national data on food consumption and changes in the diet of Brazilians from 2008 to 2018

**DOI:** 10.11606/s1518-8787.2021055supl1ap

**Published:** 2021-11-22

**Authors:** Rosely Sichieri

**Affiliations:** I Universidade do Estado do Rio de Janeiro Instituto de Medicina Social Hesio Cordeiro Departamento de Epidemiologia Rio de Janeiro RJ Brasil Universidade do Estado do Rio de Janeiro. Instituto de Medicina Social Hesio Cordeiro. Departamento de Epidemiologia. Rio de Janeiro, RJ, Brasil

Over the last 50 years, Brazilian society had major changes in its demographic structure and in the urban-rural distribution of its population, with just over 15% of Brazilians living in rural areas, according to the *Pesquisa Nacional por Amostra de Domicílios* (PNAD – National Household Sample Survey) of 2015^1^. These changes were followed by epidemiological and nutritional transitions, with a great decay in child malnutrition rates. In the 70s, malnutrition affected 56% of children aged 0 to 5 living in the Northeast and 38% of those living in the South-Southeast. These data were collected by the *Estudo Nacional da Despesa Familiar* (Endef – National Study on Household Expenditure)^2^ between 1974 and 1975. The Endef was the first and most complete study on diet in Brazil to fully assess the severity of malnutrition and the regional distribution of budgets of Brazilian families. In terms of the dietary consumption of a significant portion of the population, the data showed such a dire situation that they were not released – not even for the researchers in that subject – during the military dictatorship. Foods consumed at home were weighed for a week. However, data were compiled for each family unit, not for individuals. Since then, the *Pesquisa de Orçamentos Familiares* (POF – Household Budget Survey) is source of all knowledge about diet in Brazil, estimating consumption from food available at home within a week. That is, the POF does not assess individual consumption. For budget purposes, only foods consumed at home are counted, while outside meals are not. Even so, the POF data allow great comparisons between national and international consumption trends for households^3^.

On the other hand, ignorance about outside meals is a limitation. The POF records expenses with outside meals, but many of these data prioritize the cost of food instead of its composition. It records, as examples, expenses with snacks, meals, and beverages. Outside meals have increased worldwide and also in Brazil. In the 2008–2009 POF, 41.2% of individuals aged 10 or older reported buying food outside. This percentage was higher for men than for women and also varied with age: 46% for adults, 37.7% for adolescents and 24.2% for elders^4^.

Description of diet in Brazil was already a concern in 1946, including regional characteristics and the social and biological aspects of eating. That year, Josué de Castro published the classic “The Geography of Hunger”, introducing the concept of food areas, endemic hunger areas, epidemic hunger areas, and malnutrition areas, thus making the first map of hunger in Brazil. By classifying geographic regions according to their resources, the local diet, the availability of regional products and the health of inhabitants, it is clear that one’s biological and sociocultural characteristics are influenced by diet. Castro describes endemic hunger area as a certain geographical area in which at least half of the population has nutritional deficiencies. In addition to the pioneer Josué de Castro, other authors have tried to understand diet in Brazil and how it relates to the country’s most common diseases and public policies. Their studies are summarized by Vasconcelos, 2007^5^.

Because the Nutrition and Collective Health field has expanded, research left this broad integration of biological, geographic and sociocultural characteristics. However, systemic approaches recently combined multiple adverse conditions that interact synergistically, which explains excess morbidity in a population. In 2019, *The Lancet* published a broad analysis based on the syndemic aspects of obesity, malnutrition and climate change that involve the food production-consumption cycle^6^.

In this sense, the 2017–2018 POF^7^ includes several data sets that allow more systemic approaches to diet by combining food availability at home, food consumption, and food safety, besides the usual well defined socioeconomic data

To overcome the limits of budget research in diet analysis, the last two Brazilian POF from 2008–2009^8^ and 2017–2018^7^ introduced a new module that estimates individual diet in subsamples. This is important progress, since it not only allows assessing individuals by their characteristics, but it can also combine the two types of consumption assessment, as some studies have already done^9^.

Assessment of individual diet allows estimating if public health programs are adequate, including: 1) fortification of flour with iron^10^; 2) low impact of sodium reduction in industrialized foods^11^; 3) the risk of excessive consumption of folic acid if various dosages of supplement are used during pregnancy^12^. Thus, data from the individual consumption module called *Inquérito Nacional de Alimentação* (INA – National Food Survey) allow assessing various policies and priorities in nutrition, including those of salt, sugar, iron, and folate consumption. A periodic collection of these data can review current policies and establish limits for sodium amount in industrialized foods, as well as for flour fortification.

This supplement of the *Revista de Saúde Pública* (Public Health Journal) continues the efforts of the area of Nutrition in Public Health in the assessment of food consumption of Brazilians while addressing methodological aspects of the most recent INA, done by IBGE in a subsample of the 2017–2018 POF^7^ (IBGE, 2020). The Brazilian Ministry of Health dedicated a big effort and a generous budget to create the INA, coordinated by Eduardo Augusto Fernandes Nilson. André Luiz Martins from IBGE also devoted himself to make the individual consumption module work.

The technical staff who developed the module in 2017-2018 and conducted adequacy studies to compare it with the 2008–2009 INA was coordinated by the following teachers: Rosely Sichieri of the Institute of Social Medicine at Universidade do Estado do Rio de Janeiro (UERJ); Rosângela Alves Pereira of the Department of Social and Applied Nutrition at Universidade Federal do Rio de Janeiro (UFRJ); Edna Massae Yokoo of the Department of Epidemiology and Biostatistics at Universidade Federal Fluminense (UFF); and Dirce Maria Lobo Marchioni of the Faculdade de Saúde Pública at Universidade de São Paulo (USP).

Renata da Rocha Muniz Rodrigues of the Institute of Social Medicine at Universidade do Estado do Rio de Janeiro (UERJ) stood out for organizing various groups that assessed and corrected records with unidentified foods, tested data consistency, and analyzed them. Eduardo De Carli of the Faculdade de Saúde Pública, Universidade de São Paulo (USP), adjusted the *Tabela Brasileira de Composição de Alimentos* (TBCA – Brazilian Food Composition Table) of the Food Research Center (FoRC) for the INA. Eliana Bistriche Giuntini and other researchers from the FoRC of the Faculdade de Ciências Farmacêuticas quantified foods of all receipts reported in the 2017–2018 INA. Ilana Nogueira Bezerra of the Graduate Program in Collective Health at Universidade Federal do Ceará (UFC) adjusted the Table of Homemade Measures. Eliseu Verly Junior and Dayan Carvalho Ramos Salles de Oliveira, both from the Institute of Social Medicine, Universidade do Estado do Rio de Janeiro (UERJ), Amanda de Moura Souza of the Institute of Collective Health Studies, Universidade Federal do Rio de Janeiro (UFRJ), and Marina Campos Araújo of the Escola Nacional de Saúde Pública Sérgio Arouca, Fiocruz, made the main compatibility analysis with the 2008–2009 INA, since the two surveys had different methods for data collection and composition table. These compatibility results are shown in the methodological manuscript of the supplement.

In addition to methodological details, the supplement approaches comparisons between the two surveys on the most consumed foods and changes of energy and nutrient intake, especially those of greatest interest to public health and to the dietary patterns observed. In short, Brazilian adolescents, adults and elders kept a dietary pattern based on rice, beans, meat, and coffee, but except for coffee, these foods were not consumed as much as in the previous survey. A lower consumption of soft drinks was a very positive change brought by the 10-year gap between the two surveys, but milk and dairy were also less consumed. The higher income class had more positive changes in diet, such as increase in whole grain consumption, whereas adolescents had the worst food intake profile. Nutrient inadequacy was still dominant because of less beans, milk, cheese, meats and fruits, and low consumption of oilseeds and fish.

Thus, as for morbidity data, diets uncover the historical inequalities and inequities of Brazil. Poorer individuals showed greater prevalence of inadequate intake for most of the nutrients, same as the poorest regions of the country, North and Northeast.

Simulations on how to obtain a healthy and culturally acceptable diet between 2009 and 2018, which is analyzed by the supplement, were also more expensive in the poorer ranges. Eating healthy became much more expensive according to the latest survey.

An adequate and culturally acceptable diet is the basis of a happy and healthy life. Learning about diet in Brazil is essential to understand ourselves as a nation and to provide future generations with food security. After looking at these supplement data, we hope governments will maintain and improve surveys and regularly collect consumption data along with the budget surveys by IBGE. These data have been used in many academic research and are of major importance to propose and assess public policies that are essential in this post-pandemic period after so many changes in food consumption and general consumption.
