# Supplementary material for: Importance of national data on food consumption and changes in the diet of Brazilians from 2008 to 2018
Source: Rev Saude Publica. 2021 Nov 22;55 Supl 1:2s. doi: 10.11606/s1518-8787.2021055supl1ap (PMC9586433; doi:10.11606/s1518-8787.2021055supl1ap)
Supplement: Translation

# Translation: Importância de dados nacionais sobre o consumo alimentar e mudanças na alimentação dos brasileiros de 2008 a 2018

**Authors:** Rosely Sichieri

**Affiliations:** I Rio de Janeiro RJ Brasil Universidade do Estado do Rio de Janeiro. Instituto de Medicina Social Hesio Cordeiro. Departamento de Epidemiologia. Rio de Janeiro, RJ, Brasil

Nos últimos 50 anos, a sociedade brasileira sofreu transformações intensas na sua estrutura demográfica e na distribuição urbano-rural de sua população, com pouco mais de 15% da população vivendo em zona rural, segundo a Pesquisa Nacional por Amostra de Domicílios (PNAD) de 2015^1^. Transição epidemiológica e nutricional acompanharam essas mudanças com importante redução nas altas prevalências de desnutrição infantil. Na década de 70 do século passado, a desnutrição acometia 56 % das crianças de 0 a 5 anos vivendo no Nordeste e 38% daquelas vivendo no Sul-Sudeste. Esses dados foram coletados pelo Estudo Nacional da Despesa Familiar- Endef^2^, realizado entre 1974 e 1975. O Endef foi o primeiro e mais completo estudo sobre alimentação dos brasileiros e a primeira pesquisa a avaliar, em profundidade, a gravidade da desnutrição e a distribuição regional dos orçamentos familiares da população brasileira. Os dados mostravam uma situação tão grave em relação ao consumo alimentar de parcela importante da população, que ficaram anos sem serem liberados pela ditadura militar, mesmo para os pesquisadores estudiosos do tema. Os alimentos consumidos nos domicílios foram pesados durante uma semana, contudo os dados foram compilados para a unidade familiar e não para os indivíduos. Desde então o conhecimento sobre a alimentação no Brasil tem sido baseado nas Pesquisas de Orçamentos Familiares (POF), que permitem estimar o consumo a partir na disponibilidade domiciliar de alimentos no período de uma semana. Ou seja, a POF não permite avaliar o consumo individual e por definição para fins de orçamento, somente são computados alimentos que passam pelo domicílio, de forma que não são consideradas as refeições fora do domicílio. Mesmo assim, os dados das POF permitem ótimas comparações de tendência de consumo nacionais e internacionais para agregados de domicílios^3^.

Por outro lado, não conhecer os alimentos e refeições realizadas fora do domicílio é uma limitação. Na POF são registrados gastos com alimentação fora, mas muitos desses dados priorizam o custo da alimentação e não sua composição, por exemplo, são registrados os gastos com lanches, refeição, bebidas. A importância da alimentação fora do domicílio tem aumentado em todo mundo e também no Brasil. Na POF de 2008–2009, 41,2% dos indivíduos com 10 anos ou mais relataram compra de alimentos fora de casa. Esse percentual foi maior para homens do que para mulheres e variou também com a idade, sendo de 46% para os adultos, 37,7% para os adolescentes e de 24,2% entre os idosos^4^.

Já em 1946 havia uma preocupação por detalhar a alimentação do brasileiro, suas características regionais e as relações sociais e biológicas da alimentação. Nesse ano, ao publicar o clássico “Geografia da fome”, Josué de Castro introduz os conceitos de áreas alimentares, áreas de fome endêmica, áreas de fome epidêmica, áreas de subnutrição, e assim produz o primeiro mapa da fome no país. Ao classificar as regiões geográficas segundo os recursos existentes, a alimentação habitual, a disponibilidade de produtos regionais e a saúde da população fica claro que os habitantes dessas áreas refletem, em suas características biológicas e socioculturais, a influência marcante da dieta. Por área de fome endêmica, Castro entende uma determinada área geográfica em que pelo menos metade da população apresenta nítidas manifestações de carências nutricionais. Além do trabalho pioneiro de Josué de Castro, outros autores buscaram entender a alimentação do brasileiro e sua relação com as doenças mais prevalentes e as políticas públicas no Brasil e esses estudos estão sumarizados na revisão de Vasconcelos, 2007^5^.

Com a evolução do campo de estudo da Nutrição e da Saúde Coletiva as pesquisas evoluíram abandonando essa integração abrangente das características biológicas, geográficas e sociocultural, porém tem ocorrido, mais recentemente, abordagens sistêmicas que combinam múltiplas condições adversas que interagem de forma sinérgica explicando o excesso de morbidade em uma população. Em 2019, o periódico *The Lancet* publicou ampla análise baseada nos aspectos sindêmicos da obesidade, subnutrição e mudanças climáticas que envolvem o ciclo de produção-consumo de alimentos^6^.

Nesse sentido, a POF de 2017–2018^7^ inclui diversos conjuntos de dados que permitem abordagens mais sistêmicas da alimentação ao combinar disponibilidade do alimento no domicílio, o consumo alimentar e o capítulo da segurança alimentar, além dos clássicos dados socioeconômicos muito bem caracterizados nesse tipo de inquérito.

Para superar as limitações da pesquisa de orçamentos na avaliação do consumo alimentar, nas últimas duas POF brasileiras 2008–2009^8^ e 2017–2018^7^, foi introduzido um novo módulo que estima o consumo alimentar individual, em subamostras. Esse é um avanço importante, pois além de permitir avaliações por características dos indivíduos, as duas formas de avaliação do consumo podem ser combinadas como já tem sido feito^9^.

A avaliação do consumo alimentar individual tem permitido estimar a adequação de programas de saúde pública como: 1) a fortificação com ferro das farinha^10^; 2) o baixo impacto da redução de sódio nos alimentos industrializados pactuada com a indústria^11^; 3) o risco de excesso de consumo de ácido fólico com uso de várias dosagens de suplemento durante a gestação^12^. Assim, os dados do Inquérito Nacional de Alimentação (INA), como tem sido chamado o módulo de consumo individual, permitem avaliar várias políticas e prioridades em nutrição, como as relativas ao consumo de sal, açúcar, ferro e folato. A coleta periódica desses dados permite a revisão de políticas, por exemplo, o estabelecimento de metas de sódio para alimentos industrializados, bem como para a fortificação das farinhas.

O presente suplemento da Revista de Saúde Pública dá continuidade aos esforços da área de Nutrição em Saúde Pública na avaliação do consumo alimentar do brasileiro cobrindo aspectos metodológicos do mais recente INA, conduzido pelo IBGE em uma subamostra da POF 2017–2018^7^. Vale salientar o esforço e recursos orçamentários do Ministério da Saúde para que fosse possível a execução do INA, coordenado pelo Eduardo Augusto Fernandes Nilson, e pelo IBGE a importante dedicação do André Luiz Martins Costa na viabilização do módulo de consumo individual.

A equipe técnica que desenvolveu o módulo em 2017–2018 e realizou estudos de adequação para comparações com o INA de 2008–2009 foi coordenada pelas professoras: Rosely Sichieri do Instituto de Medicina Social da UERJ, Rosângela Alves Pereira do Departamento de Nutrição Social e Aplicada da UFRJ, Edna Massae Yokoo do Departamento de Epidemiologia e Bioestatística da UFF e Dirce Maria Lobo Marchioni da Faculdade de Saúde Pública da USP.

Destacou-se na organização dos diferentes grupos de trabalho que avaliaram os registros com alimentos não identificados, fizeram a correção dos mesmos, testaram a consistência dos dados, e participaram da análise a pesquisadora Renata da Rocha Muniz Rodrigues do Instituto de Medicina Social, UERJ. Eduardo De Carli da Faculdade de Saúde Pública, USP fez a adequação da Tabela Brasileira de Composição de Alimentos (TBCA) do Centro de Pesquisa em Alimentos da Universidade de São Paulo para o INA e Eliana Bistriche Giuntini e demais pesquisadores do Food Research Center – FoRC, da Faculdade de Ciências Farmacêuticas desenvolveram a quantificação de alimentos de todas as receitas das preparações informadas no INA de 2017–2018. Ilana Nogueira Bezerra do Programa de Pós-Graduação em Saúde Coletiva, UFC fez a adequação da Tabela de Medidas Caseiras. Eliseu Verly Junior e Dayan Carvalho Ramos Salles de Oliveira, ambos do Instituto de Medicina Social, UERJ, Amanda de Moura Souza do Instituto de Estudos em Saúde Coletiva, UFRJ e Marina Campos Araújo da Escola Nacional de Saúde Pública Sergio Arouca, Fiocruz fizeram as principais análises de compatibilização com o INA de 2008–2009, dado que o método de coleta de dados e tabela de composição foram diferentes nos dois inquéritos. Esses resultados de compatibilização estão apresentados no manuscrito metodológico que compõem o suplemento.

Além dos detalhes metodológicos, o suplemento reúne os resultados referentes às comparações entre os dois inquéritos dos alimentos mais consumidos, a evolução da ingestão de energia e de nutrientes, particularmente aqueles de maior interesse para a Saúde Pública e os padrões alimentares observados. Em síntese, pode-se dizer que a alimentação de adolescentes, adultos e idosos brasileiros manteve um padrão de consumo à base de arroz, feijão, carne e café, porém com redução importante do consumo desses itens, exceto para o café. Redução do consumo de refrigerantes foi um aspecto bem positivo das mudanças no período de 10 anos entre os dois inquéritos, mas houve também redução importante de leite e laticínios. As mudanças positivas na alimentação foram mais acentuadas na maior classe de renda, como o aumento no consumo de grãos integrais e mudanças para pior sempre foram mais acentuadas entre os adolescentes. Continuaram expressivas as altas prevalências de inadequação de nutrientes, compatível com a redução de feijão, leite, queijos, carnes e frutas e baixo consumo de oleaginosas e peixes.

Assim, como para dados de morbidade, o consumo alimentar desvenda as históricas desigualdades e iniquidades do Brasil. Os indivíduos com menor renda apresentaram as maiores prevalências de inadequação para a maioria dos nutrientes investigados, assim como as regiões mais pobres do país, Norte e Nordeste, apresentaram no geral maiores prevalências de inadequação no consumo de nutrientes.

Os modelos de simulação para obtenção de uma alimentação saudável e culturalmente aceitável entre os períodos de 2009 e 2018, objeto de uma das análises do suplemento, também mostraram um maior custo nas menores faixas de renda. Na análise temporal, obter uma alimentação mais saudável tornou-se muito mais caro no inquérito mais recente.

A alimentação adequada e culturalmente aceitável é a base de uma vida feliz e saudável, conhecer os vários aspectos da alimentação dos brasileiros é um passo fundamental para nos conhecermos como nação e para prover o futuro das gerações com segurança alimentar. Nós que nos debruçamos sobre esses dados do suplemento esperamos que os governos mantenham e aprimorem os inquéritos, coletando periodicamente dados de consumo com as pesquisas de orçamento pelo IBGE. Esses dados têm sido fonte de inúmeras pesquisas acadêmicas e importante base para propor e avaliar políticas públicas tão fundamentais nessa conjuntura pós-pandemia de tantas mudanças em todos os aspectos do consumo e, em particular, do consumo alimentar.
